# Supplementary material for: High-performance and stable photoelectrochemical water splitting cell with organic-photoactive-layer-based photoanode
Source: Nat Commun. 2020 Nov 2;11:5509. doi: 10.1038/s41467-020-19329-0 (PMC7606446; doi:10.1038/s41467-020-19329-0)
Supplement: Supplementary file 1 — Supplementary Information [file 41467_2020_19329_MOESM1_ESM.pdf]

Supplementary Information

High-performance and stable photoelectrochemical water splitting cell with organic-photoactive-layer-based photoanode

Je Min Yu[1,6], Jungho Lee[1,2,6], Yoon Seo Kim[3,6], Jaejung Song[3], Jiyeon Oh[1], Sang Myeon Lee[1], Mingyu Jeong[1], Yongseon Kim[1], Ja Hun Kwak[1], Seungho Cho[3,*], Changduk Yang[1,4] *, and Ji-Wook Jang[1,5] *

[1] School of Energy and Chemical Engineering, Ulsan National Institute of Science and Technology (UNIST), Ulsan 44919, Republic of Korea.

[2] Department of Chemistry, Purdue University, West Lafayette, IN 47907, USA

[3] School of Materials Science and Engineering, Ulsan National Institute of Science and Technology (UNIST), Ulsan 44919, Republic of Korea.

[4] School of Energy and Chemical Engineering, Perovtronics Research Center, Low Dimensional Carbon Materials Center, Ulsan National Institute of Science and Technology (UNIST), Ulsan 44919, Republic of Korea

[5] Emergent Hydrogen Technology R&D Center, Ulsan National Institute of Science and Technology (UNIST), Ulsan 44919, Republic of Korea

[6] These authors contributed equally to this work: Je Min Yu, Jungho Lee, and Yoon Seo Kim

*Email: jiwjang@unist.ac.kr (J.-W.J.); yang@unist.ac.kr (C.Y.); scho@unist.ac.kr (S.C.)

Supplementary figures

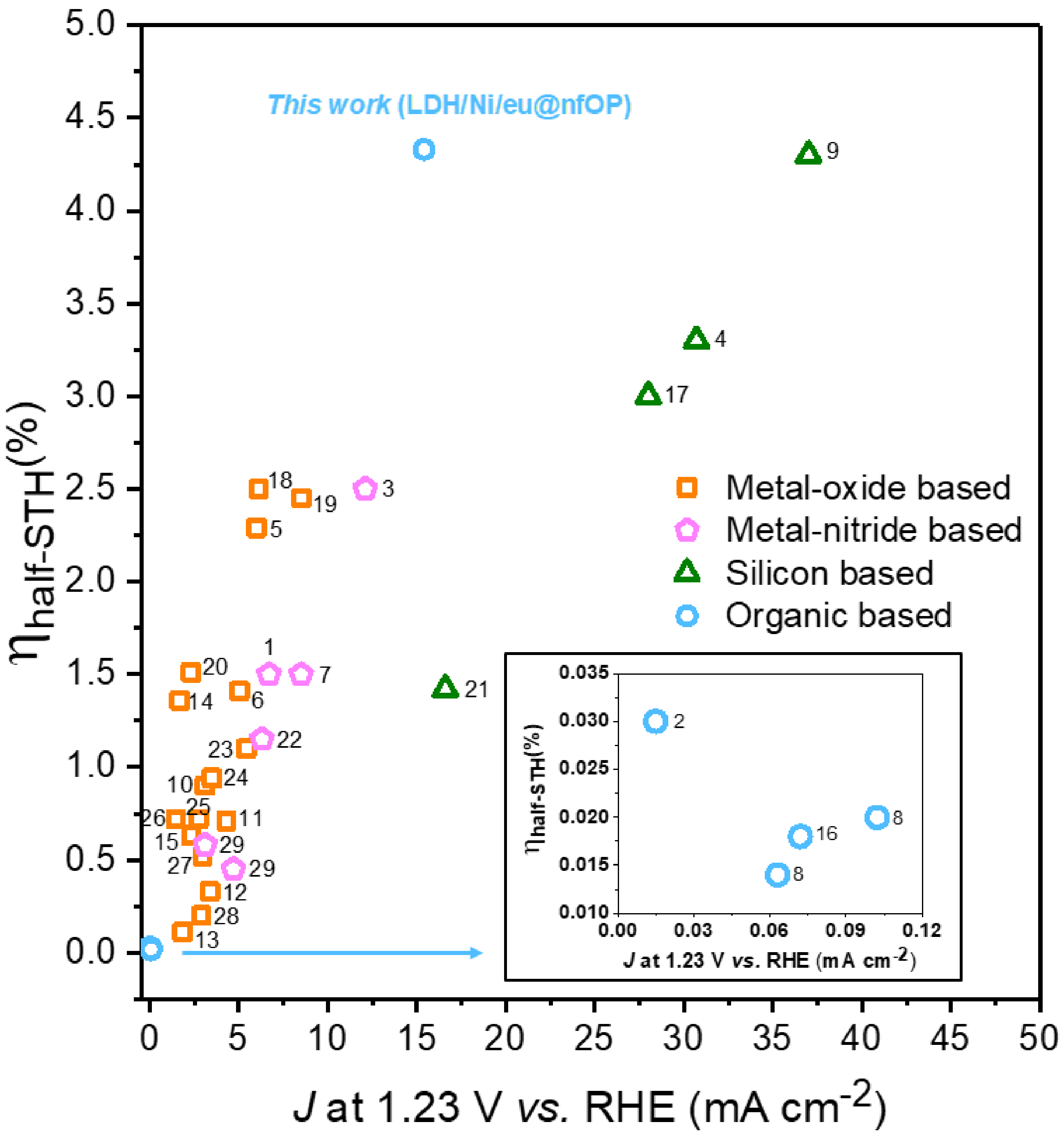

Supplementary Fig. 1 | Recent reported half-cell solar-to-hydrogen conversion efficiencies ($\eta_{half-STH}$) and photocurrent density at 1.23 V vs. reversible hydrogen evolution of photoanodes.

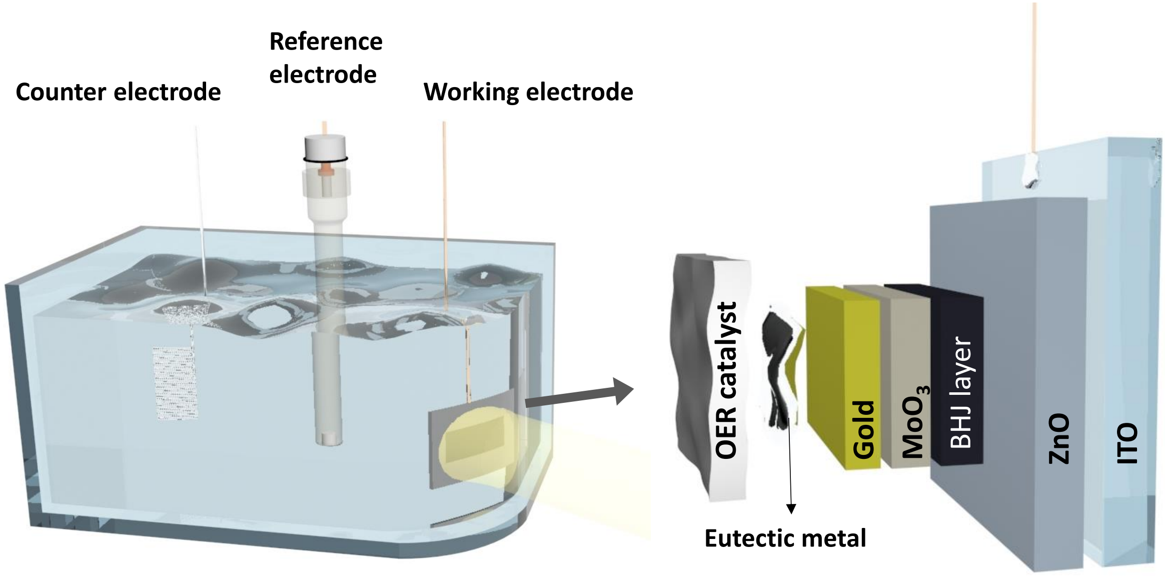

Supplementary Fig. 2 | Overall schematic of organic-photoactive-layer-based photoanode.

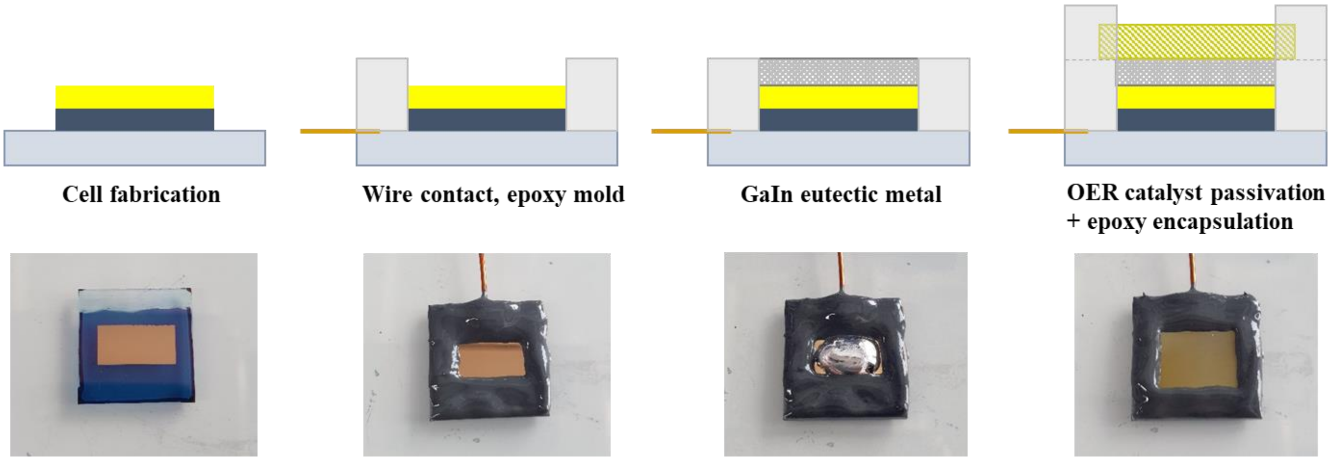

Supplementary Fig. 3 | Schematic and photographic images of organic photoanode fabrication process. First, silver paste and epoxy were used to connect a copper wire on the prepared organic-photoactive-layer-based cell. Second, after applying GaIn eutectic, NiFe-LDH/Ni foil was loaded on the electrode. Finally, an epoxy bond was applied to fix NiFe-LDH/Ni foil and encapsulate the electrode.

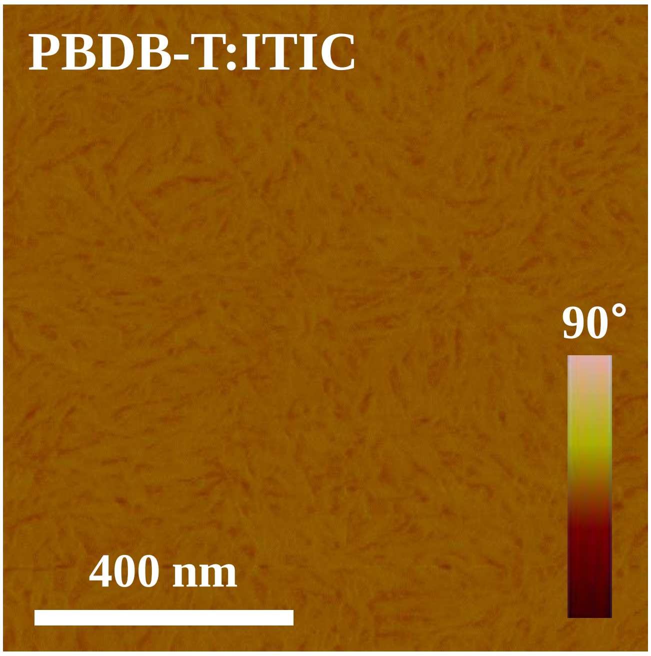

Supplementary Fig. 4 | AFM phase image of PBDB-T:ITIC-based OPVs.

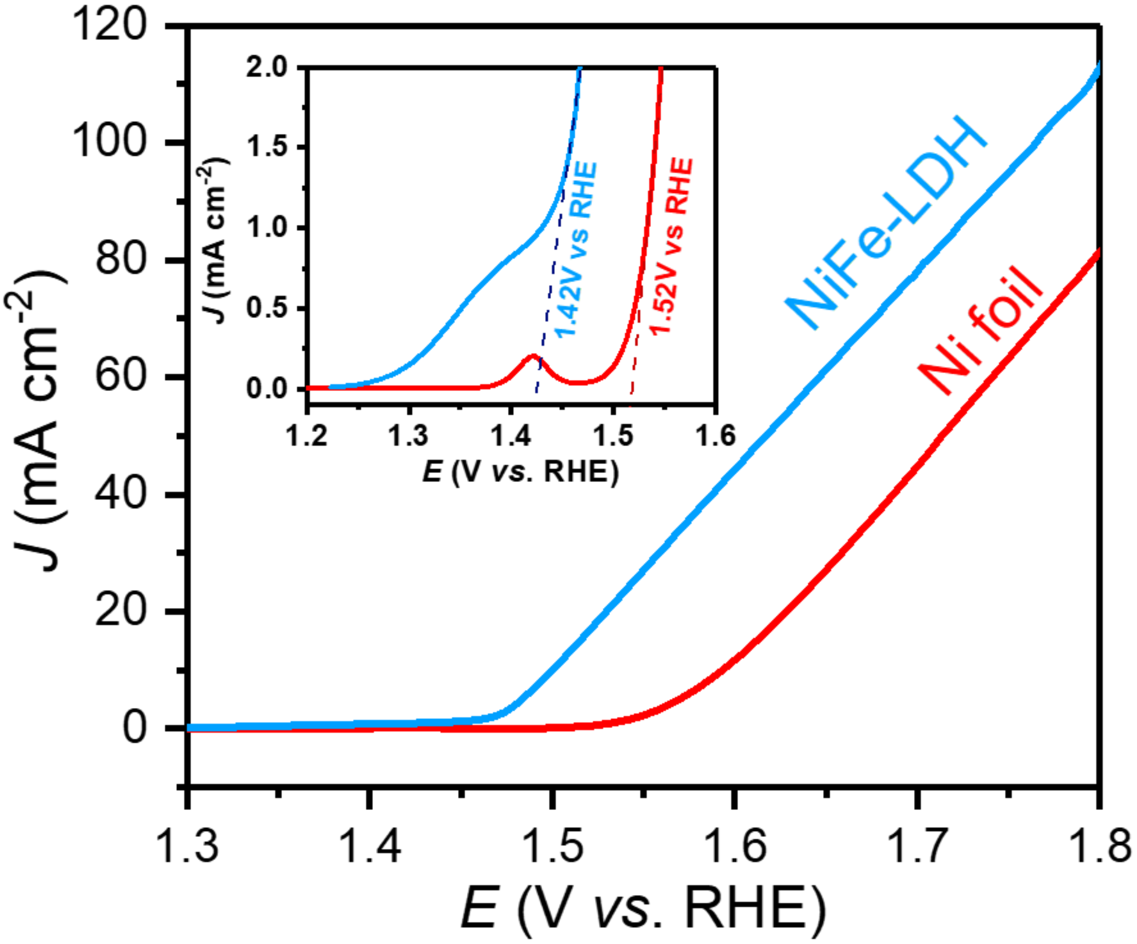

Supplementary Fig. 5 | Electrochemical performance of Ni foil and NiFe-LDH-loaded Ni electrode.

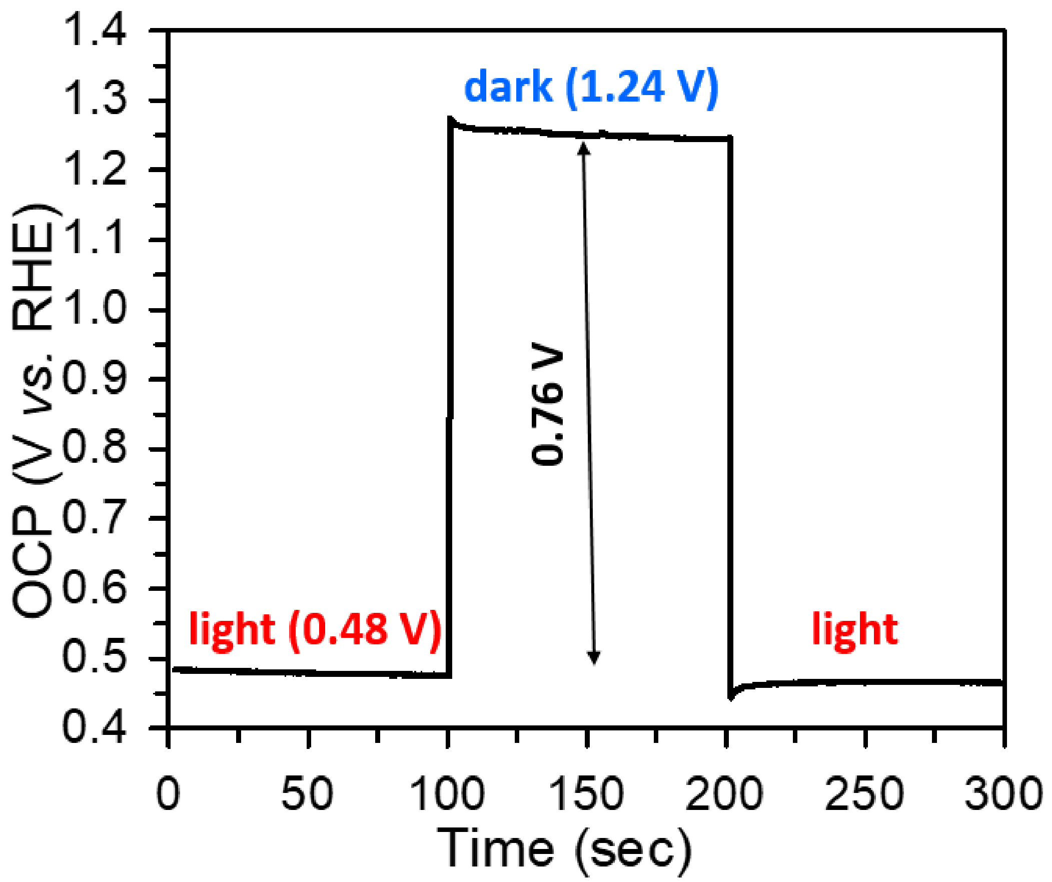

Supplementary Fig. 6 | Open-circuit potential measurement using LDH/Ni/eu@nfOP under light and dark conditions (1 M NaOH, AM 1.5G illumination).

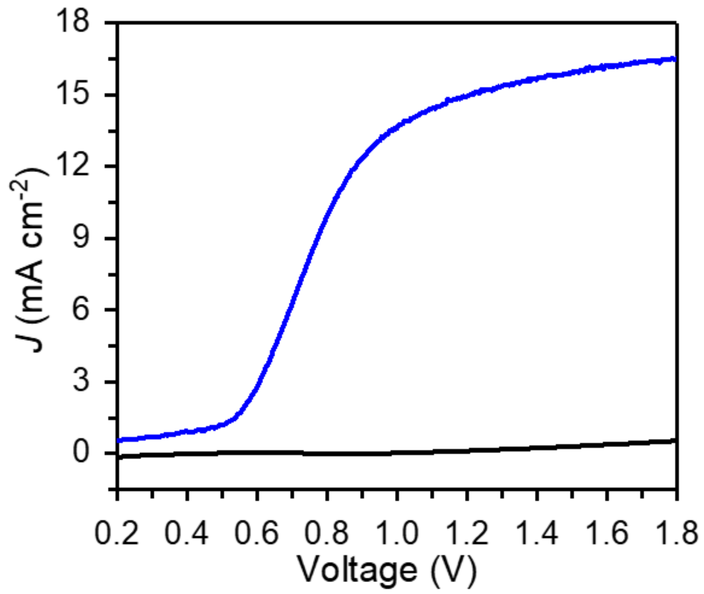

Supplementary Fig. 7 | Current density–potential of LDH/Ni/eu@nfOP in a two-electrode system.

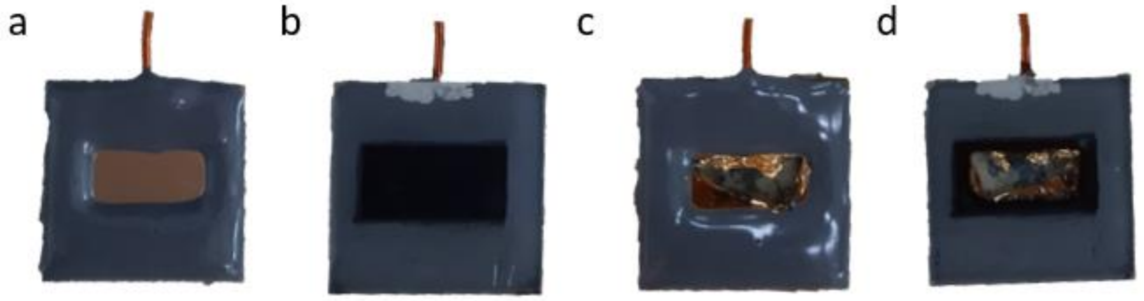

Supplementary Fig. 8 | Photographic images of nfOP. a, Back side of pristine nfOP. **b,** Front side of pristine nfOP. **c,** Back side of nfOP. **d,** Front side of nfOP after the PEC performance measurement.

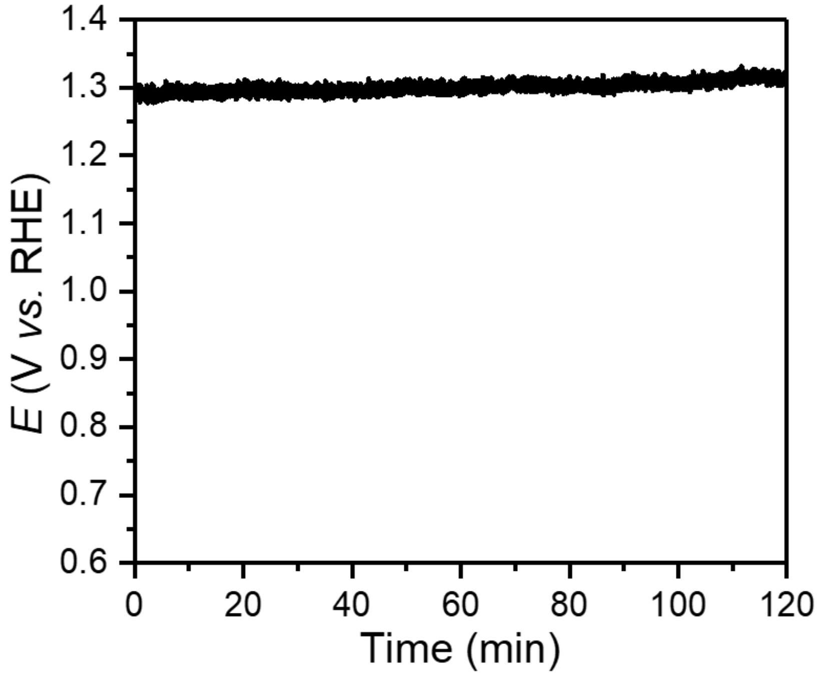

Supplementary Fig. 9 | The potential–time curve of LDH/Ni/eu@nfOP at 15.4 mA cm^{-2} in 1M NaOH under AM 1.5G illumination.

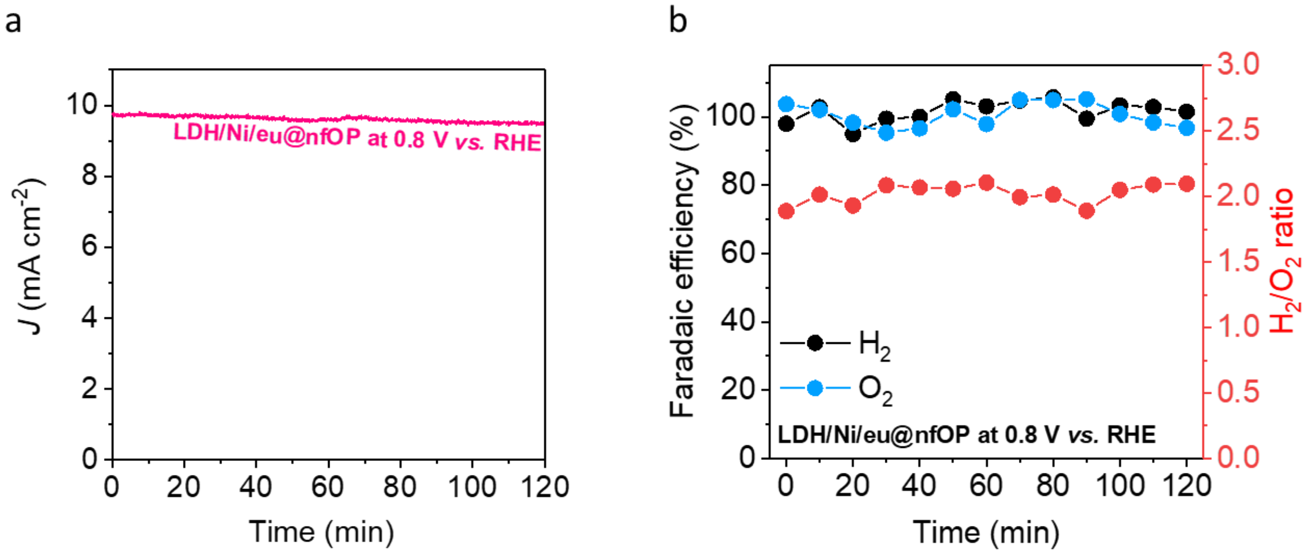

Supplementary Fig. 10 | The photoelectrochemical water splitting at highest $\eta_{half-STH}$ potential. a, Current density – time curves of LDH/Ni/eu@nfOP at 0.8 V *vs.* RHE for water oxidation in 1 M NaOH under AM 1.5 G illumination. **b**, Faradaic efficiencies for H_2 and O_2 production and H_2/O_2 ratio for LDH/Ni/eu@nfOP at the same condition.

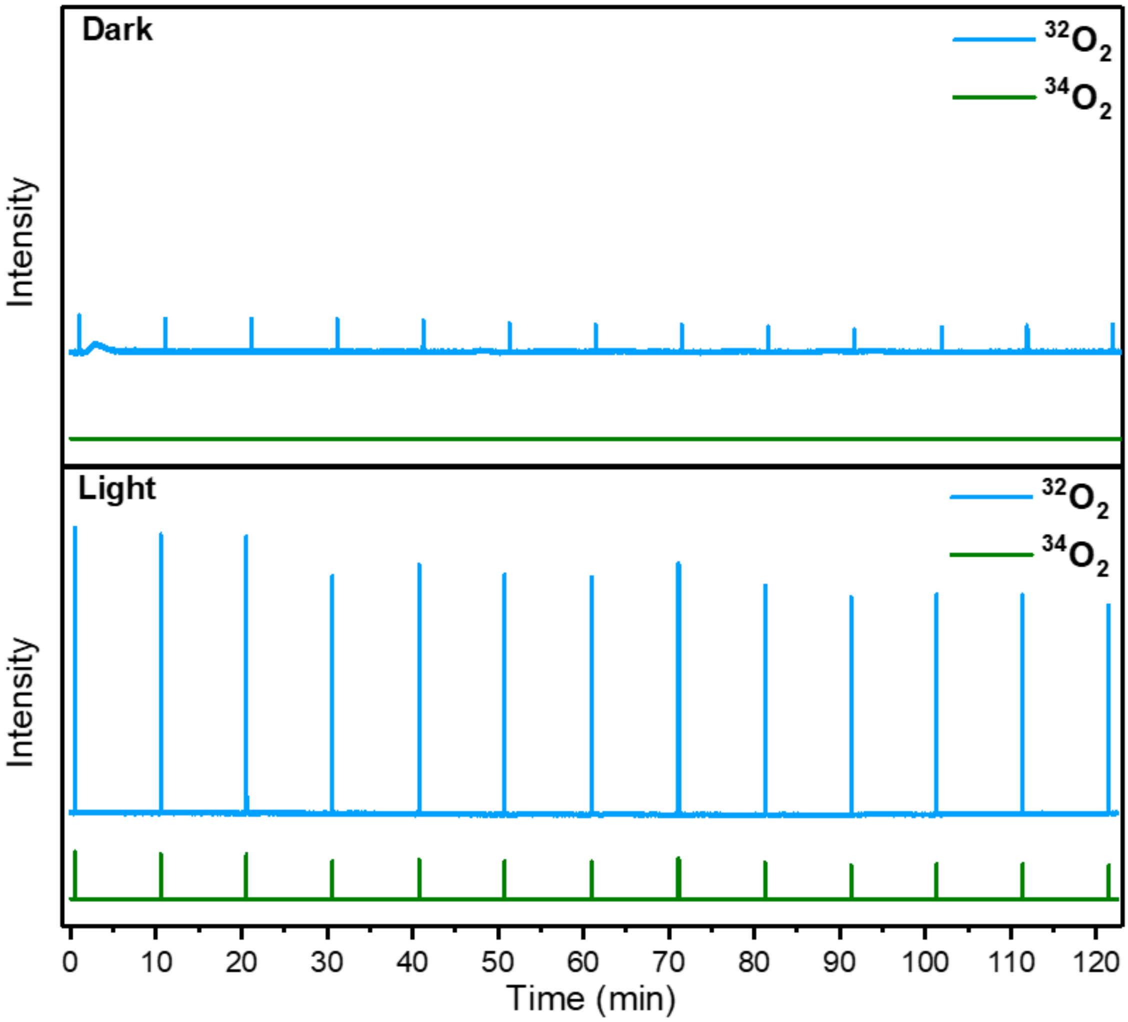

Supplementary Fig. 11 | Mass spectra of $^{32}O_2$ and $^{34}O_2$ evolution of LDH/Ni/eu@nfOP in ^{18}O-labelled H_2O (10 %) added 1 M NaOH under dark and AM 1.5G illumination.

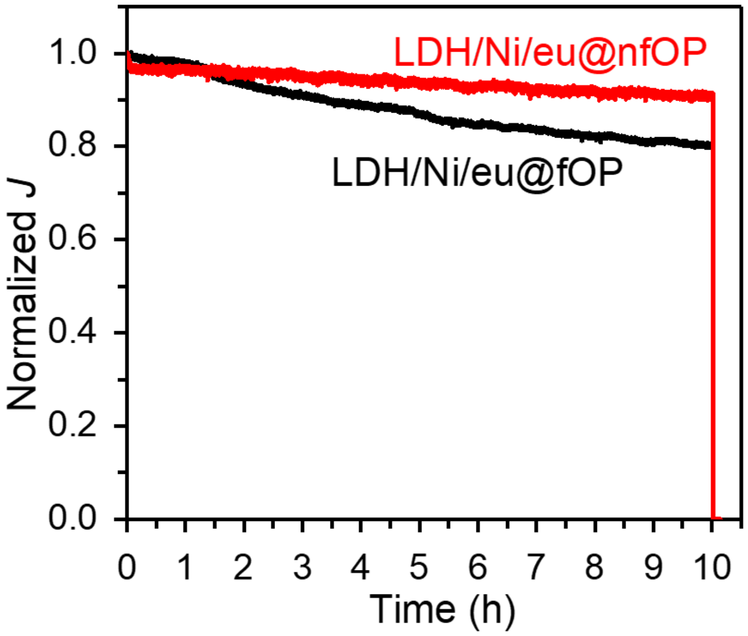

Supplementary Fig. 12 | Comparison of normalized current density−time curves of LDH/Ni/eu@nfOP and LDH/Ni/eu@fOP in 1 M NaOH under AM 1.5G illumination. (fOP:PBDB-T:$PC_{71}BM$-based OPVs)

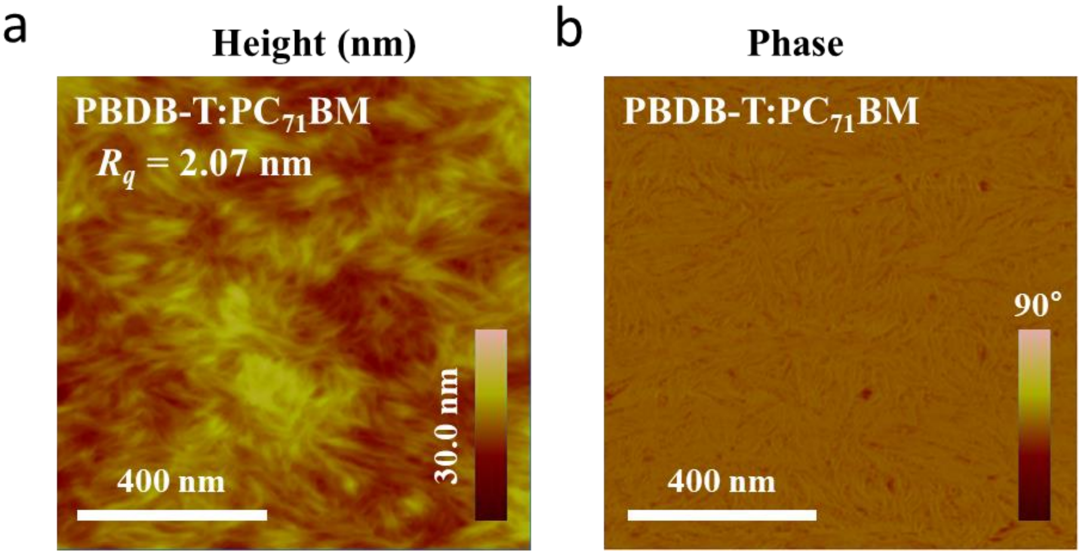

Supplementary Fig. 13 | AFM image of PBDB-T:PC_{71}BM-based OPVs. a, AFM height image and **b,** AFM phase image of PBDB-T:PC_{71}BM.

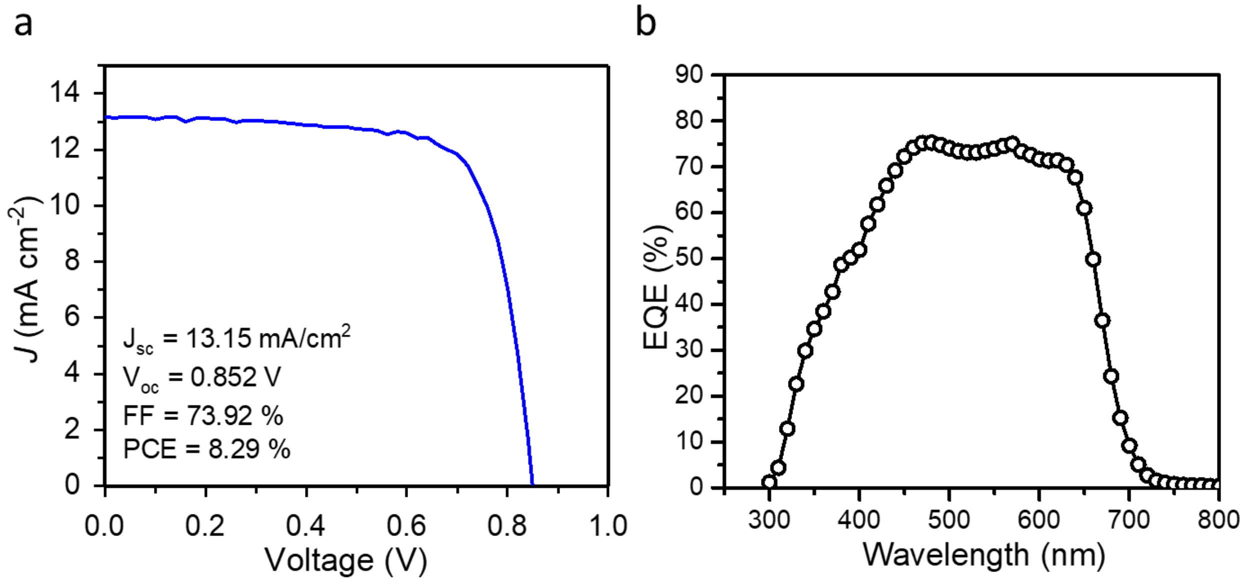

Supplementary Fig. 14 | Performance of PBDB-T:PC$_{71}$BM-based OPVs. a, Current density−voltage curve of PBDB-T:PC$_{71}$BM-based OPVs under AM 1.5G solar simulator (100 mW cm^{-2}). **b,** EQE for the PBDB-T:PC$_{71}$BM OPV.

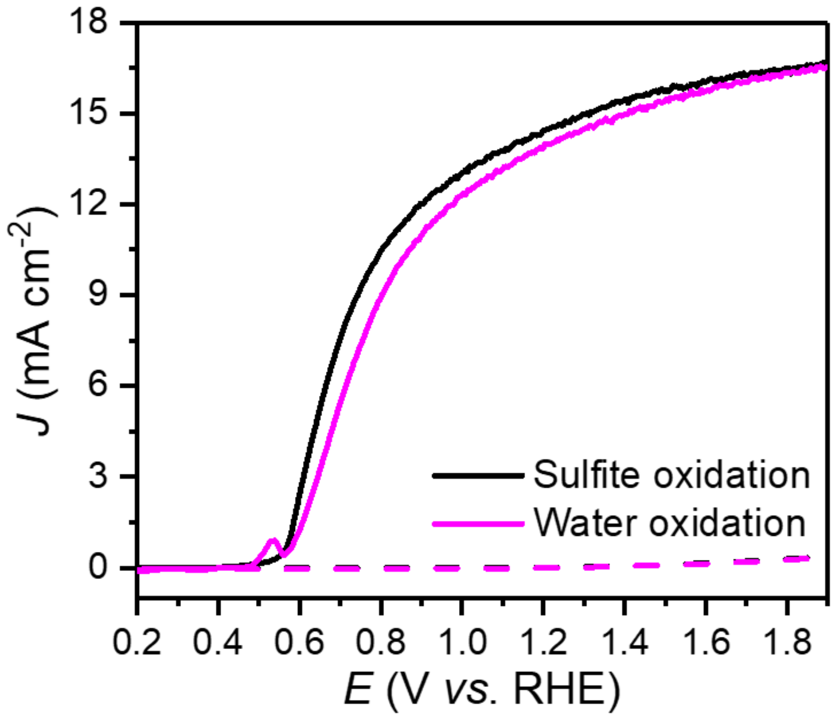

Supplementary Fig. 15 | Comparison of current density–potential curves of LDH/Ni/eu@nfOP with and without sulfite under AM 1.5G illumination (100 mW cm^{-2}). For the sulfite oxidation, we added 0.4 M Na_2SO_3 to 1 M NaOH electrolyte.

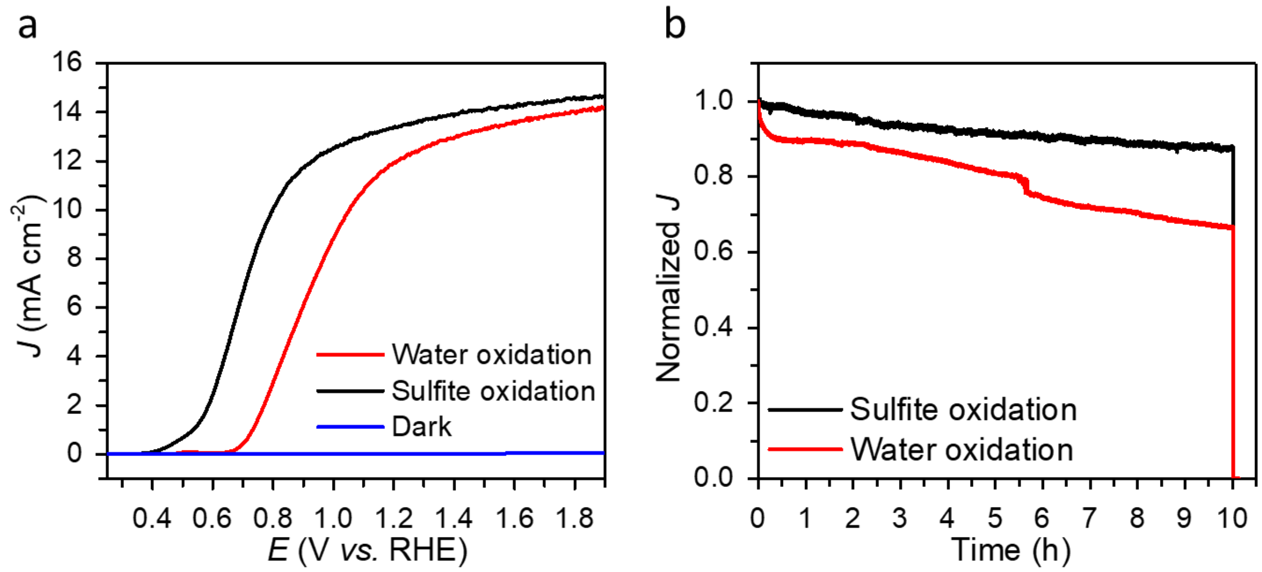

Supplementary Fig. 16 | Comparison of performances of Ni/eu@nfOP with and without sulfite under AM 1.5G illumination (100 mW cm^{-2}). a, Comparison of current density–potential curves for water oxidation and sulfite oxidation. **b,** Comparison of normalized current density–time curves for water oxidation and sulfite oxidation**.** Sulfite oxidation condition was measured in 1 M NaOH containing 0.4 M Na_2SO_3 and water oxidation was measured in 1 M NaOH.

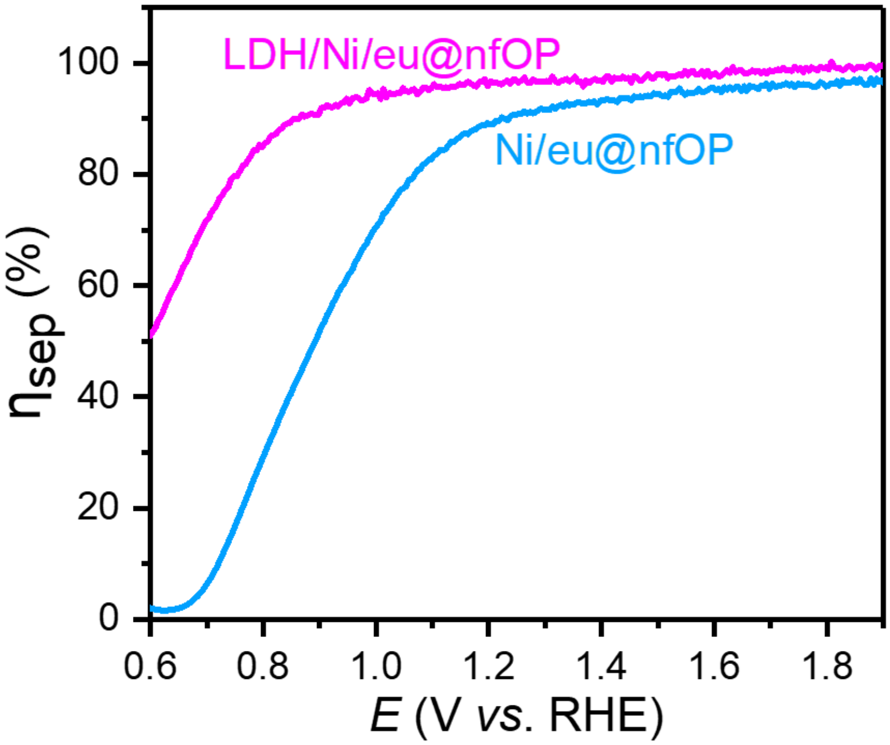

Supplementary Fig. 17 | Comparison of surface charge separation efficiency of Ni/eu@nfOP and LDH/Ni/eu@nfOP under AM 1.5G illumination (100 mW cm^{-2}).

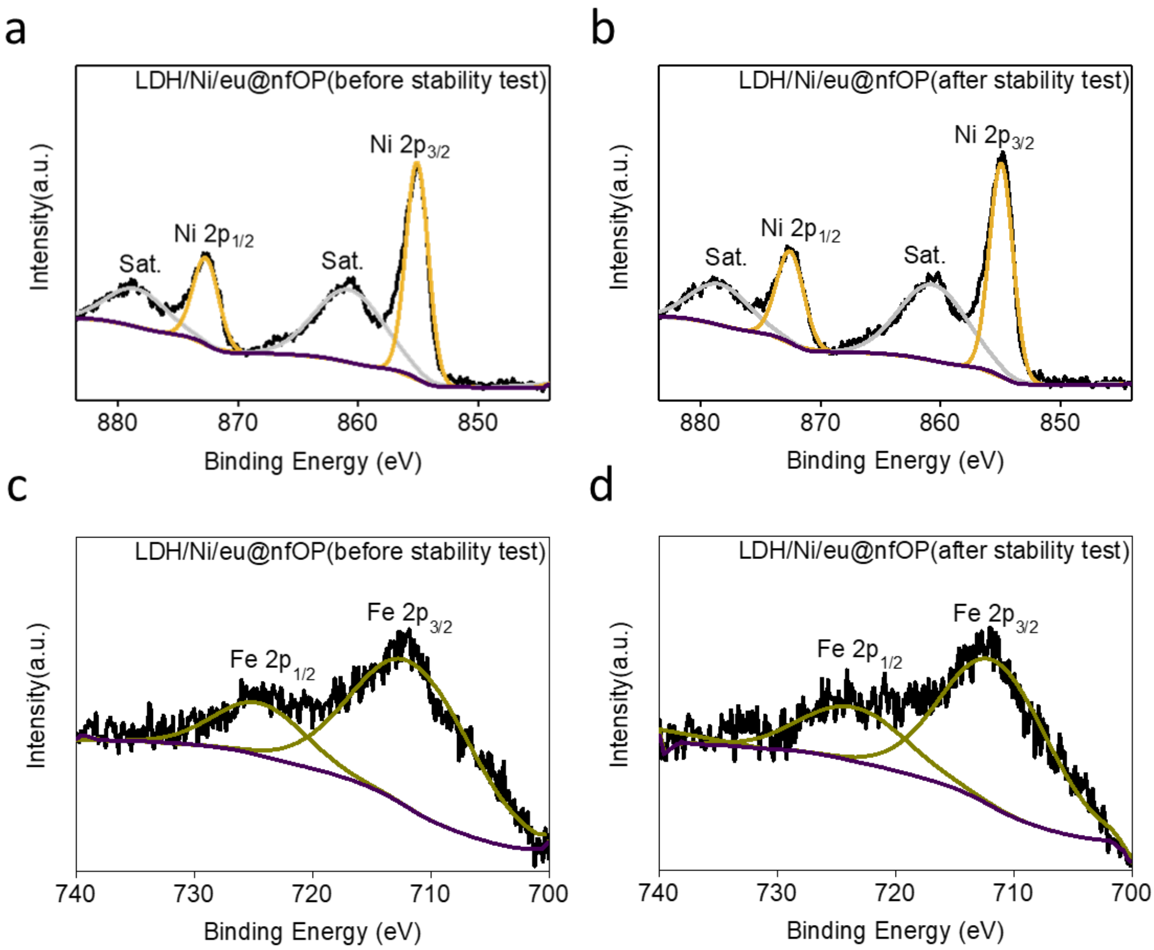

Supplementary Fig. 18 | High-resolution XPS spectrum of NiFe-LDH/Ni/eu@nfOP. a, b, Ni 2p, **c, d,** Fe 2p in LDH/Ni/eu@nfOP before and after 10 h stability test, respectively.

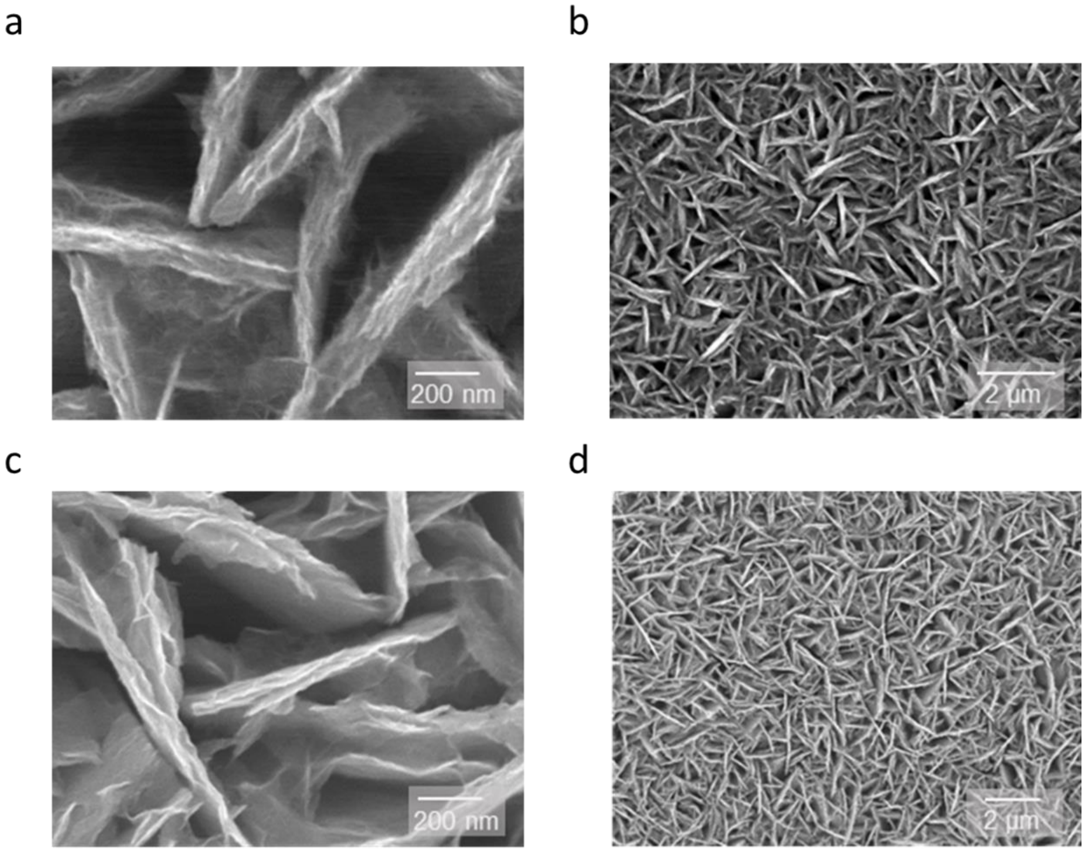

Supplementary Fig. 19 | SEM images of LDH/Ni/eu@nfOP. a,b, Surface of LDH/Ni/eu@nfOP before and **c,d,** after 10 h stability test.

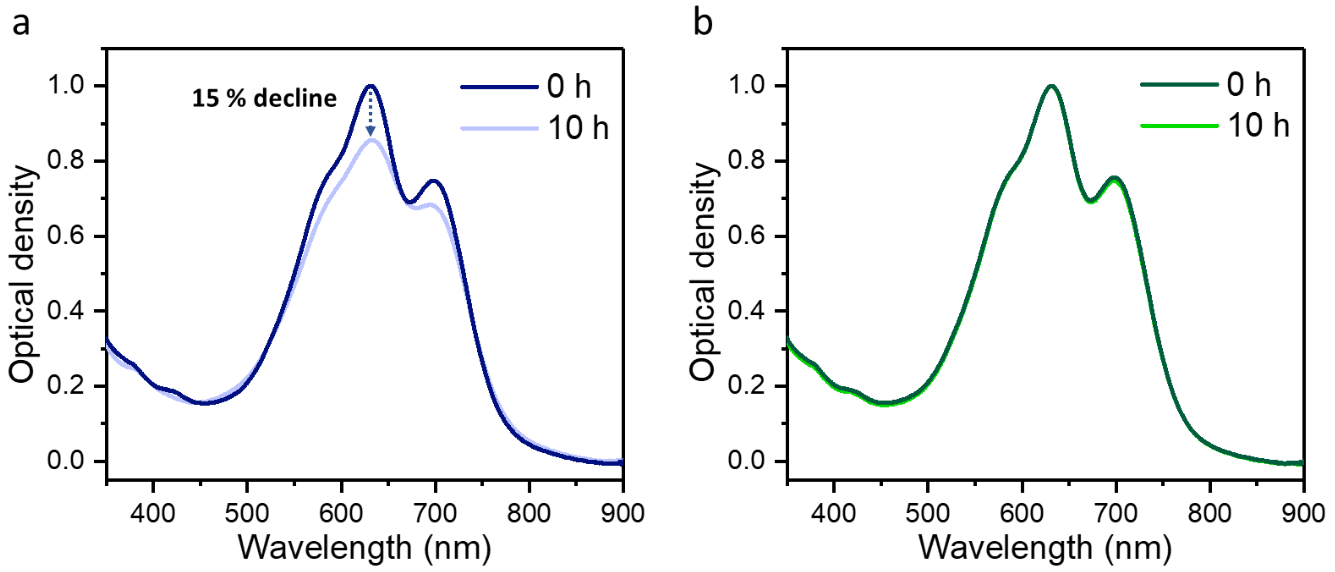

Supplementary Fig. 20 | Comparison of UV-Vis absorption spectra of PBDB-T:ITIC-based BHJ layer before and after 10 h light irradiation. a, Under irradiation of AM 1.5G illumination (UV + Vis) light. **b**, Under irradiation of only Vis light.

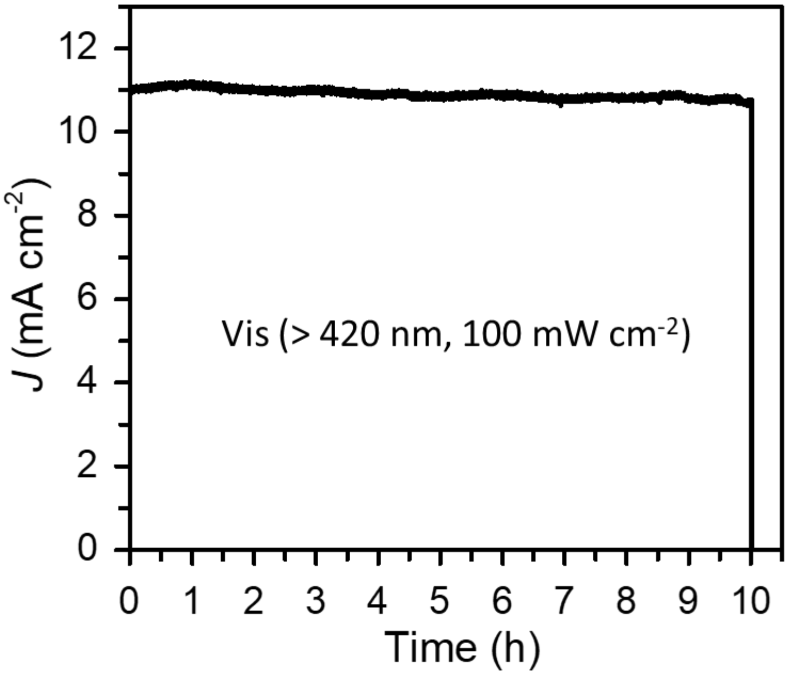

Supplementary Fig. 21 | Current density–time curves of LDH/Ni/eu@nfOP at 1.3 V *vs*. RHE under Vis-light illumination with 420 nm cut-off filter (100 mW cm^{-2}).

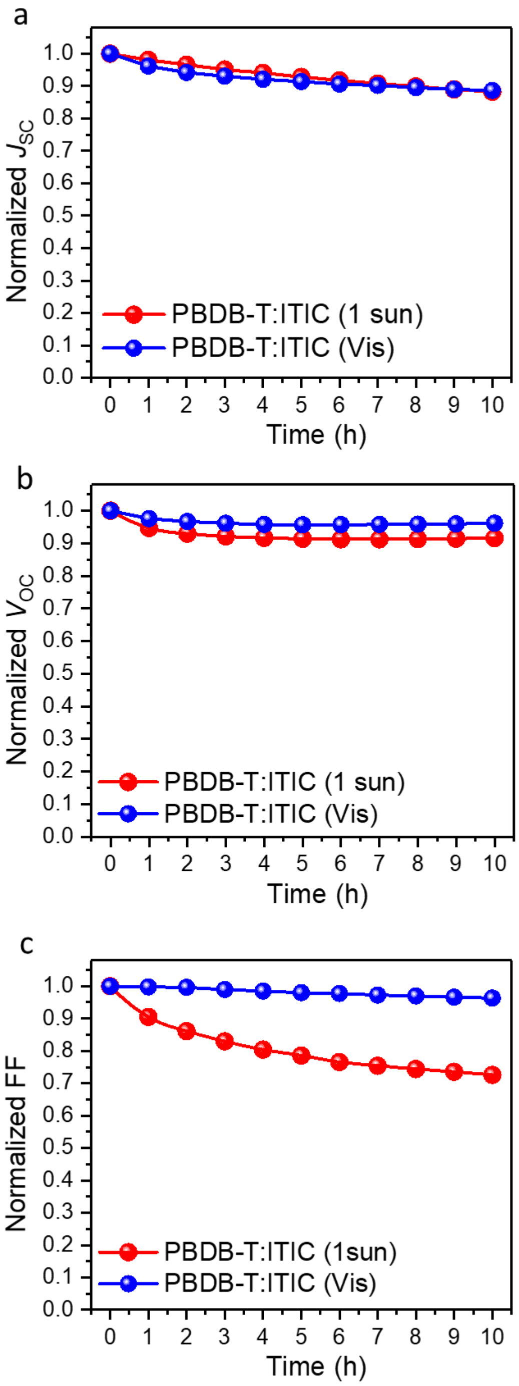

Supplementary Fig. 22 | Comparison of normalized performance–time curve of PBDB-T:ITIC-based OPVs under AM 1.5G illumination and Vis-light illumination with 420 nm cut-off filter. a, Comparison of normalized short-circuit current (J_{sc})–time curve. **b,** Comparison of normalized open-circuit voltage (V_{oc})–time curve. **c,** Comparison of normalized FF–time curve.

Supplementary Table. 1 | Recent reported PEC performance of photoanodes.

Year	Photoanode	Onset potential (V *vs.* RHE)	*J* at 1.23 V *vs.* RHE (mA cm^{-2})	$\eta_{half-STH}$ (%)	Electrolyte	Ref
2013	Co-Pi/Ba-Ta_3N_5	0.65	6.7	1.5%	0.5 M K_2HPO_4 (pH 13)	(1)
2015	BBL	< 0.7	0.015	~0.03%	0.5 M Na_2SO_4 (pH 7)	(2)
2016	Integrated Ta_3N_5(P)	0.6	12.1	2.5%	1 M NaOH (pH 13)	(3)
2017	np$^+$-Si/SiO_x/NiO_x/NiFe	0.89	30.7	3.30%	1M KOH (pH 13.7)	(4)
2017	NiOOH-FeOOH/CQDs/BiVO4	-	5.99	2.29%	potassium phosphate (pH 7)	(5)
2017	CoO_x coated Mo,N-$BiVO_4$ on Ti substrate	-	5.04	1.41%	0.1M phosphate buffer (pH 7.4)	(6)
2017	CoPi/GaN/Ta_3N_5	0.65	-	1.50%	0.5M potassium phosphate (pH 13)	(7)
2017	g-C_3N_4	0.5	0.063	~0.014%	0.1 M Na_2SO_4 (pH 7)	(8)
2017	s-BCN	0.5	0.1023	~0.02%	0.1 M Na_2SO_4 (pH 6.6)	(8)
2018	NiFe-LDH onto the polycrystalline n$^+$p-Si substrate	0.78	37	4.30%	1M KOH	(9)
2018	[Co_2(bim)$_4$]- modified $BiVO_4$	-	3.1	0.90%	0.5 M Na_2SO_4.	(10)
2018	β- FeOOH/$BiVO_4$	-	4.3	0.71%	0.2 M Na_2SO_4 (pH 7)	(11)
2018	Ti:Fe_2O_3@GCNN-CQDs	-	3.38	0.33%	1 M KOH (pH 13.6)	(12)
2018	CoOOH/(Ti, C) - Fe_2O_3	-	1.85	0.11%	NaOH (pH 14)	(13)
2018	surface modificated TiO_2-B/anatase core/shell NWs	-	1.69	1.36%	1M NaOH	(14)
2018	red-colored polymerized C_3N_4 on TiO_2 nanorod	-	2.33	0.63%	1M Na_2SO_4	(15)
2018	rGO/g-C_3N_4	0.8	0.072	~0.018%	0.1 M KOH	(16)
2019	n-Si/SiO_x/Al_2O_3/Ni/NiO_x/NiOOH	0.85	28	3%	1M KOH	(17)
2019	black $BiVO_4$@TiO_{2-x}	-	6.12	2.50%	0.5M potassium phosphate (pH 7)	(18)
2019	Mo-$BiVO_4$@rGO composite	0.19	8.51	2.45%	0.1M Na_2SO_4 (pH 7)	(19)
2019	Ti^{3+}/Ni co-doped TiO_2 nanotube	-	-	1.51%	1M KOH	(20)
2019	n-Si/CoOx/$NiCuO_x$	1.04	16.6	1.42%	1M NaOH	(21)
2019	$NiFeO_x$/Ta_3N_5/GaN/Al_2O_3	0.65	6.3	1.15%	0.2M potassium phosphate (pH 13)	(22)

2019	F/Mo:$BiVO_4$ with CoPi	-	4.78(F) / 5.43(B)	1.10%	0.1M KH_2PO_4/K_2HPO_4 buffer (pH 7.3)	(23)
2019	Ultrathin $Co(OH)_x$ encap p-Cu_2S/n-$BiVO_4$	-	3.51	0.94%	0.5M potassium phosphate (pH 10)	(24)
2019	NiO/$BiVO_4$	-	2.75	0.72%	0.1M Potassium phosphate (pH 7.4)	(25)
2019	$BiFeO_3$ coated Sn:TiO_2 (BFO/Sn:TiO_2)	0.18	1.47	0.72%	1M NaOH	(26)
2019	CoPi onto Mo:$BiVO_4$	-	2.98	0.52%	0.1M phosphate buffer (pH 7)	(27)
2019	Fe_2O_3/TiO_2	-	2.9	0.20%	1M KOH	(28)
2020	Ta_3N_5-NRs/$BaTaO_2N$/$FeNiO_X$	>0.6	4.7	0.45%	0.5 M K_2HPO_4 (pH 13)	(29)
2020	Ta_3N_5-NRs/$FeNiO_X$	0.6	3.1	0.58%	0.5 M K_2HPO_4 (pH 13)	(29)
**This work**	**LDH/Ni/eu@nfOP**	**0.55**	**15.1**	**4.33%**	**1M NaOH (pH 13.6)**	

Supplementary References

1 Li, Y. *et al.* Cobalt phosphate-modified barium-doped tantalum nitride nanorod photoanode with 1.5% solar energy conversion efficiency. *Nat. Commun.* **4**, 2566 (2013).

2 Bornoz, P., Prévot, M. S., Yu, X., Guijarro, N. & Sivula, K. Direct light-driven water oxidation by a ladder-type conjugated polymer photoanode. *J. Am. Chem. Soc.* **137**, 15338-15341 (2015).

3 Liu, G. *et al.* Enabling an integrated tantalum nitride photoanode to approach the theoretical photocurrent limit for solar water splitting. *Energy Environ. Sci.* **9**, 1327-1334 (2016).

4 Yu, X., Yang, P., Chen, S., Zhang, M. & Shi, G. NiFe alloy protected silicon photoanode for efficient water splitting. *Adv. Energy Mater.* **7**, 1601805 (2017).

5 Ye, K.-H. *et al.* Carbon quantum dots as a visible light sensitizer to significantly increase the solar water splitting performance of bismuth vanadate photoanodes. *Energy Environ. Sci.* **10**, 772-779 (2017).

6 Gu, J. *et al.* In situ growth of a TiO_2 layer on a flexible Ti substrate targeting the interface recombination issue of $BiVO_4$ photoanodes for efficient solar water splitting. *J. Mater. Chem. A* **5**, 20195-20201 (2017).

7 Zhong, M. *et al.* Highly active GaN-stabilized Ta_3N_5 thin-film photoanode for solar water oxidation. *Angew. Chem., Int. Ed.* **56**, 4739-4743 (2017).

8 Ruan, Q. *et al.* A nanojunction polymer photoelectrode for efficient charge transport and separation. *Angew. Chem., Int. Ed.* **56**, 8221-8225 (2017).

9 Guo, B. *et al.* Facile integration between Si and catalyst for high-performance photoanodes by a multifunctional bridging layer. *Nano Lett.* **18**, 1516-1521 (2018).

10 Zhang, W. *et al.* A cobalt-based metal–organic framework as cocatalyst on $BiVO_4$ photoanode for enhanced photoelectrochemical water oxidation. *ChemSusChem* **11**, 2710-2716 (2018).

11 Zhang, B., Wang, L., Zhang, Y., Ding, Y. & Bi, Y. Ultrathin FeOOH nanolayers with abundant oxygen vacancies on $BiVO_4$ photoanodes for efficient water oxidation. *Angew. Chem., Int. Ed.* **57**, 2248-2252 (2018).

12 Yi, S.-S., Yan, J.-M. & Jiang, Q. Carbon quantum dot sensitized integrated Fe_2O_3@g-C_3N_4 core–shell nanoarray photoanode towards highly efficient water oxidation. *J. Mater. Chem. A* **6**, 9839-9845 (2018).

13 Ye, K.-H. *et al.* A novel CoOOH/(Ti, C)-Fe_2O_3 nanorod photoanode for photoelectrochemical water splitting. *Sci. China Mater.* **61**, 887-894 (2018).

14 Tian, Z. *et al.* Highly conductive cable-like bicomponent titania photoanode approaching limitation of electron and hole collection. *Adv. Funct. Mater.* **28**, 1803328 (2018).

15 Yang, Y. *et al.* An unusual red carbon nitride to boost the photoelectrochemical performance of wide bandgap photoanodes. *Adv. Funct. Mater.* **28**, 1805698 (2018).

16 Peng, G., Volokh, M., Tzadikov, J., Sun, J. & Shalom, M. Carbon nitride/reduced graphene oxide film with enhanced electron diffusion length: An efficient photo-electrochemical cell for hydrogen generation. *Adv. Energy Mater.* **8**, 1800566 (2018).

17 Luo, Z. *et al.* Multifunctional nickel film protected n-type silicon photoanode with high photovoltage for efficient and stable oxygen evolution reaction. *Small Methods*, 1900212 (2019).

18 Tian, Z. *et al.* Novel black $BiVO_4$/TiO_{2-x} photoanode with enhanced photon absorption and charge separation for efficient and stable solar water splitting. *Adv. Energy Mater.* **9**, 1901287 (2019).

19 Subramanyam, P., Vinodkumar, T., Nepak, D., Deepa, M. & Subrahmanyam, C. Mo-doped $BiVO_4$@reduced graphene oxide composite as an efficient photoanode for photoelectrochemical water splitting. *Catal. Today* **325**, 73-80 (2019).

20 Dong, Z., Ding, D., Li, T. & Ning, C. Facile preparation of Ti^{3+}/Ni co-doped TiO_2 nanotubes photoanode for efficient photoelectrochemical water splitting. *Appl. Surf. Sci.* **480**, 219-228 (2019).

21 He, L. *et al.* Cascading interfaces enable n-Si photoanodes for efficient and stable solar water oxidation. *J. Phys. Chem. Lett.* **10**, 2278-2285 (2019).
22 Higashi, T. *et al.* Transparent Ta_3N_5 photoanodes for efficient oxygen evolution toward the development of tandem cells. *Angew. Chem., Int. Ed.* **58**, 2300-2304 (2019).
23 Rohloff, M. *et al.* Enhanced photoelectrochemical water oxidation performance by fluorine incorporation in $BiVO_4$ and Mo:$BiVO_4$ thin film photoanodes. *ACS Appl. Mater. Interfaces* **11**, 16430-16442 (2019).
24 He, B. *et al.* Spatial engineering of a $Co(OH)_x$ encapsulated p-Cu_2S/n-$BiVO_4$ photoanode: simultaneously promoting charge separation and surface reaction kinetics in solar water splitting. *J. Mater. Chem. A* **7**, 6747-6752 (2019).
25 Huang, Q. *et al.* p-type NiO modified $BiVO_4$ photoanodes with enhanced charge separation and solar water oxidation kinetics. *Mater. Lett.* **249**, 128-131 (2019).
26 Huang, J. *et al.* Synergistically enhanced charge separation in $BiFeO_3$/Sn:TiO_2 nanorod photoanode via bulk and surface dual modifications. *Nano Energy* **59**, 33-40 (2019).
27 Liu, C. *et al.* Cobalt–phosphate-modified Mo:$BiVO_4$ mesoporous photoelectrodes for enhanced photoelectrochemical water splitting. *J. Mater. Sci.* **54**, 10670-10683 (2019).
28 Feng, F. *et al.* Boosting hematite photoelectrochemical water splitting by decoration of TiO_2 at the grain boundaries. *Chem. Eng. J.* **368**, 959-967 (2019).
29 Pihosh , Y. *et al.* Development of a core–shell heterojunction Ta_3N_5-nanorods/$BaTaO_2N$ photoanode for solar water splitting. *ACS Energy Lett.* **5**, 2492-2497 (2020).
